# An assessment of the barriers to accessing the Basic Package of Health Services (BPHS) in Afghanistan: was the BPHS a success?

**DOI:** 10.1186/s12992-016-0212-6

**Published:** 2016-11-15

**Authors:** Alexandra Frost, Matthew Wilkinson, Peter Boyle, Preeti Patel, Richard Sullivan

**Affiliations:** 1 Centre for Global Health, King’s Health Partners and King’s College London, London, UK; 2 Conflict and Health Research Group, King’s College London, London, UK; 3 Department of War Studies, King’s College London, London, UK; 4 Centre of Islamic Studies, SOAS, University of London, London, UK; 5 International Prevention Research Institute, France and University of Strathclyde Institute of Global Public Health @iPRI, Lyon, France

**Keywords:** Health system reconstruction, Fragile states, Health system policy, Aid effectiveness, Maternal health, Health system financing, Afghanistan

## Abstract

Afghanistan is one of the most fragile and conflict-affected countries in the world. It has experienced almost uninterrupted conflict for the last thirty years, with the present conflict now lasting over a decade. With no history of a functioning healthcare system, the creation of the Basic Package of Health Services (BPHS) in 2003 was a response to Afghanistan’s dire health needs following decades of war. Its objective was to provide a bare minimum of essential health services, which could be scaled up rapidly through contracting mechanisms with Non-Governmental Organisations (NGOs). The central thesis of this article is that, despite the good intentions of the BPHS, not enough has been done to overcome the barriers to accessing its services. This analysis, enabled through a review of the existing literature, identifies and categorises these barriers into the three access dimensions of: acceptability, affordability and availability. As each of these is explored individually, analysis will show the extent to which these barriers to access are a critical issue, consider the underlying reasons for their existence and evaluate the efforts to overcome these barriers. Understanding these barriers and the policies that have been implemented to address them is critical to the future of health system strengthening in Afghanistan.

## Background

Afghanistan’s history is blighted by conflict, a repetitive cycle of externally and internally driven warfare with only brief periods of peace. The decades of conflict decimated much of the social infrastructure including the country’s health system. By 2002, Afghanistan had some of the poorest health indicators of any country in the world particularly in the areas of infant, child and maternal mortality [1]. According to surveys conducted in 2002 by UNICEF and the U.S. Centers for Disease Control and Prevention (CDC) maternal mortality rates were estimated at 1,600 per 100,000 live births while United Nations Development Programme (UNDP) estimated that in 2002 under-five mortality was 257 per 1,000 live births [2, 3]. Geographical access to health services was also poor as only 10 % of the population lived within one hour’s walking distance of a health facility [4]. In response to these immense health system challenges the Basic Package of Health Services (BPHS) was launched in 2003 followed by the Essential Package of Hospital Services (EPHS) in 2005 by the Ministry of Public Health (MOPH) of the new Afghan interim government with support from international donors and non-governmental organisations (NGOs) [5]. The World Bank, the European Union and United States Agency for International Development (USAID) have been the three largest donors of the BPHS and the EPHS since their inception [6]. Collectively, they invested more than $820 million in the health sector between 2003 and 2008/09 which is particularly high when compared with investments in health sectors of other post-conflict countries [7, 8]. Each of the three main donors funded the BPHS in roughly a third of Afghanistan’s 34 provinces [9]. From 2004 to 2008 the donors provided financial and technical support, with the majority of the construction funding coming from USAID, for the renovation of 451 health facilities and the construction of 312 health facilities [10]. The BPHS and the EPHS are delivered using a contacting-out model through which local and international NGOs bid for health service contracts which last between 12 and 36 months and are designed to fulfil a standardised package of care [11]. The rapid scale up of the BPHS has been attributed to the successful implementation of the NGO contracting mechanism [9]. The BPHS is delivered by NGOs in 31 of the 34 Afghan provinces and by the MOPH in three provinces using a contracting-in initiative entitled Strengthening Mechanisms (SM) [12].

The BPHS was designed to provide key primary health services to tackle the most urgent health problems for the majority rural population [5]. It has become the favoured strategy for some post-conflict states and had already been utilised in Bosnia and Herzegovina, Cambodia, Rwanda and Uganda [1]. The package was adapted for the needs of the Afghani population and was designed to tackle the priority areas of maternal and newborn health, child health, public nutrition, and communicable diseases [13]. This was further expanded in 2005 to include disability and mental health services [11]. In addition to defining the primary health care services that are provided the BPHS also specifies the organisation and delivery of these services [7]. The package has a semi-hierarchal structure with a Health Post (HP) at the bottom, followed by a Health Sub-Centre, a Basic Health Centre, a Comprehensive Health Centre, and then the District Hospital (DH) at the top, each designed to cover a specific range of population [5]. The BPHS is available to all Afghanis and the MOPH has stipulated that everyone who needs care much receive it, regardless of their ability to pay [14]. User fees at BPHS facilities were officially banned for all Afghanis by the MOPH in April 2008 following the results of a health financing pilot study conducted between 2005 and 2007 [15]. The pilot study found that a utilisation of curative care services increased when user fees were removed [15].

The Essential Package of Hospital Services (EPHS) was developed to complement the BPHS [1]. The link between the BPHS and EPHS health services is the district hospital, which serves as the first referral-level hospital for primary care facilities [1]. The EPHS identifies a standardized package of hospital services for each level of hospital: district, provincial, regional and speciality [14]. With the two packages, the MOPH specified all the services, staffing and equipment expected at every level of the Afghan health system [1].

The BPHS has been lauded as a success in improving health indicators and for its contribution to overall health system strengthening since its introduction in 2003 [1, 7, 16]. However, there are still massive inequalities in access to healthcare across the country and between groups. For example, the majority of the country lives in rural areas (75.5 % of the total population), however, they have a lower coverage of health services and smaller ratio of health workers [17]. Urban areas have 36 health workers per 10,000 people, compared to 16.7 workers per 10,000 people in rural areas, and the most qualified professional healthcare workers [14]. Since the drawdown of international troops in 2012 violence has been escalating in post-transition Afghanistan. The United Nations Assistance Mission in Afghanistan (UNAMA) reported that 2015 was the worst year for violence against civilians since 2009, the year they started systematically documenting causalities, with women and children being particularly badly affected [18]. Increasing levels of insecurity reduces the availability of healthcare and limits civilian access to essential health services [19].

There are growing humanitarian needs in Afghanistan and it has been suggested that the health system is unable to meet these challenges [20]. Afghanistan is considered to be one of the least developed countries in the world. With a population of 31.3 million in 2014, it had a Human Development Index (HDI) of 0.465 and an HDI ranking of 171 out of 187 nations [21]. Over half of the population are considered to be in multidimensional poverty (58.8 %) and 88.1 % of those in employment earn less than $2 per day [21]. The median age of the population is 17 and 4.8 % of the population are under 5 years of age [21].

A growing body of literature has been looking at the successes and failures of the BPHS in achieving health outcomes and scaling up services [1, 22–24]. However, the barriers to accessing healthcare, in particular those related to gender, security, finance and geography, have received far less attention. This paper contributes to the field of health care strengthening in post-conflict settings by considering the barriers to accessing healthcare faced by the Afghani people and reflecting on how this has changed over time since the introduction of the BPHS in 2003. The barriers are grouped into the three access dimensions of acceptability, affordability and availability. The critical analysis that follows will consider these access dimensions individually, examining the extent to which they affect access to healthcare services and the reasons behind their existence in order to understand what can be done to achieve Universal Health Coverage (UHC) in Afghanistan [25].

## Main text

### Methods

A global literature review of both peer reviewed and grey literature was conducted in 2016 to assess the barriers to accessing the BPHS for civilians in Afghanistan. Peer reviewed studies in English from the start of the BPHS to this year (2003–2016) were sought, using the following search terms: Afghanistan AND health.

Search sites for peer reviewed literature included Google scholar, PubMed, Web of Science, the Cochrane database and Scopus. Grey literature written in English was sought from Google as well as a range of international and national sources, including the MOPH, the World Bank, the World Health Organisation, and USAID (Table 1). References from included studies were also checked to identify other relevant studies.

**Table 1:** List of websites searched

Name of organisation	Website address
Ministry of Public Health Afghanistan (MOPH)	https://moph.gov.af/en
World Bank	www.worldbank.org/
World Health Organisation	www.who.int/
USAID	https://www.usaid.gov/
UNFPA – United Nations Population Fund	www.unfpa.org/
UNDP – United Nations Development Fund	www.undp.org/
Gallup	http://www.gallup.com/home.aspx
UN Women	http://www.unwomen.org/en
Afghanistan Analysts Network	https://www.afghanistan-analysts.org/
Médecins San Frontières (MSF)	http://www.msf.org/
International NGO Safety Organisation	http://www.ngosafety.org/
International Committee of the Red Cross	https://www.icrc.org/en
Transparency International	https://www.transparency.org/

Studies that focused on the health of military personnel or on health services in other countries were excluded and the remaining studies were categorised into the three access dimensions of acceptability, affordability and availability using the definitions below. Fifty-two articles, reports or surveys were included in the final analysis, twenty-four of these were peer reviewed and twenty-eight were grey literature.

This was a non-systematic review so there was no formal assessment of bias. However, an overview of research methods reveals that approaches are heterogeneous across studies including one systematic review, one qualitative study, five literature reviews, and 17 observational studies. Studies that undertake primary data collection are limited by the security situation so frequently conduct small scale surveys or interviews. Studies that utilise secondary data collection are usually reliant on government data that has come under criticism for lack of plausibility [9]. The overall quality of evidence is therefore taken to be low.

### Theoretical frameworks for understanding barriers to accessing health care

Health care access barriers play a vital role in understanding the health inequalities and disparities that exist in a population [26]. There is a general lack of consensus in the literature about how to define access and the number and nature of dimensions to access [27]. Access is about ‘enabling a patient in need to receive the right care, from the right provider, at the right time, in the right place, dependent on context’ [28]. Several frameworks have been designed to evaluate access to healthcare [29]. The Penchansky and Thomas framework employs five dimensions: Availability, Accessibility, Accommodation, Affordability and Acceptability [30]. In this paper we have used a modified version of the Penchansky and Thomas framework focusing on the three access dimensions of availability, affordability and acceptability. These issues are interrelated but distinct and allow us to examine the key priorities of the BPHS such as improving maternal and child health and improving availability and access for the rural poor, and interventions such as the user fee ban. This modified version has been employed by Thiede et al. [27] in a study of health care in Ghana and our study is based on their definitions of these access dimensions. Analysis of access using a modified version of the Penchansky and Thomas framework is commonplace in the literature and the variation we are using appears frequently in the literature [27, 29, 31].

### Definitions of the access dimensions

**Acceptability:** the perception and nature of health service provision. Influenced by the way health services are delivered and whether they accommodate patients’ beliefs and sensitivities. Includes respect of cultural and religious beliefs, patients’ perceptions of effectiveness and sensitivity of services, patients’ readiness to engage with health professionals [27].

**Affordability:** the ‘degree of fit’ between the cost of using health care services and whether an individual can pay. Linked with the broader field of health care financing. Include factors such as health care costs, direct and indirect costs, out-of-pocket payments, household wealth and impact on household livelihoods [27].

**Availability:** are appropriate health services available in the place and time they are needed. Includes issues around location of and distance to health facilities, transportation options, mobile services, opening and waiting times and the relationship between the type, range, quantity and quality of health services provided [27].

## Results and discussion

### Acceptability

Improving maternal and child health is one of the key priorities of the BPHS. Before the implementation of the BPHS, Afghanistan had the second highest maternal mortality rate (MMR) in the world [32]. The highest ever documented maternal mortality ratio was recorded in the Badakshan province of Afghanistan between 1999 and 2002 [33]. Health services targeting women and children in Afghanistan must be sensitive to religious and cultural barriers that prevent women from accessing services. For example, it is culturally unacceptable in many areas of Afghanistan for a female patient to be seen by a male health professional [32]. Despite an overall increase in the number of female health professionals since the introduction of the BPHS there is still an overall shortage. This is one of the primary reasons for women not being able to access services. The data on the absolute number of women in the health workforce varies, according to Newbrander et al. [1] 80 % of facilities have female health staff. However, there is still unmet need and they have a particularly low presence in rural areas [34]. It was estimated that in 2012, only 23 % of the need for workforce time spent on maternal and neonatal health was met and with current graduation and attrition rates this will fall to 8 % by 2030 [35]. Cultural barriers mean that education is often not an option for women and girls, only 12 % of Afghani women are literate compared to a national literacy rate of 26 %, which prevents a new generation of female health professionals from coming to the fore [36]. Gender inequality in Afghanistan remains high with a score of 0.693 on the UNDP ‘Gender Inequality Index’ placing it 171 out of 188 countries in the world [37].

The majority of maternal deaths in Afghanistan occur due to haemorrhage and obstructed labour which are problems that can be improved with access to appropriate health services [32]. In rural Afghanistan there are cultural barriers to women seeking antenatal and intrapartum care, such as the belief that birth is a natural process that should not require external help [38]. When the BPHS was introduced in 2003, only 14.3 % of births were attended by a skilled birth attendant and only 12.8 % of births occurred at a health facility [39]. This was considered to be one of the leading contributing factors to Afghanistan’s very high maternal mortality rate. The Ministry of Public Health, with support from the international community, revitalised midwifery education in Afghanistan and focused on increasing the number of female health providers and strengthening the cadre of midwives [35]. Akseer et al., [39] found that by 2011–12 antenatal care visits, skilled birth attendance and facility-based birth had more than tripled. While these improvements are significant the more remote and inaccessible provinces report little to no increase in skilled birth attendance [39].

Family planning services are a component of the BPHS and one of the most effective interventions to reduce maternal mortality [17]. However, in practice there are large disparities in service provision. The utilisation of family planning services is low (23 % nationwide), and even lower in rural areas [17]. Roberts et al. [13] suggest that the provision of sexual and reproductive health services may be undermined by conservative political, religious and cultural entities. At the contracting level, a faith-based NGO that is awarded a BPHS contract may not provide family planning services that go against their religious values and, therefore, people may not have access to the full extent of sexual and reproductive health services to which they are entitled [13].

Another primary goal of the BPHS is to ensure increased outpatient service utilisation by women. According to studies that analysed data from the Balanced Scorecard there was an overall rise in the number of females as a percentage of new outpatients. This went from 55.2 % in 2004 to 58.0 % in 2006 (*p* < 0.01) [24], another study found that between 2004 and 2008 there was a 4.9 % increase (*p* < 0.0001) [23]. Results from the Balanced Scorecard showed that patient satisfaction and patient perceptions of quality had fluctuated between 2004 and 2008 but had remained over 75 % median for both categories, however, overall improvement was minimal [23].

#### Have there been any attempts to overcome these barriers?

There is much the Afghan state has done, in principle, to advancing the status of women, beginning with the signing of the Bonn Agreement (2001) which commits to a fully representative government responsive to issues affecting women. Since 2001 Afghanistan has also become a signatory of the Convention on the Elimination of Discrimination against Women [40] and the Millennium Declaration which promotes gender equality [41]. A ground breaking Law on the Elimination of Violence Against Women was passed in 2009, however, enforcement of this and other treaties has been weak [42]. There were a number of physical assaults against high profile women in 2013 highlighting the dangers that women in Afghanistan still face [42].

Training female health workers has been a key investment of the BPHS, with the MOPH pledging to train 10,000 additional community health workers, half of which will be women [43]. Although the pledge to train these workers is politically reassuring, it is unclear whether it has been actioned, no deadline is given in the original document and there is no suggestion of where the 14 million USD will come from to fund the project [43]. The same report states that 2200 midwives had been trained before 2010, with 1000 more to be trained between 2012 and 2014, with further claims that efforts so far have been a success [43]. There is a high attrition rate of health professionals and therefore any estimate of success given in government documents is very likely to be overly optimistic [9]. Hiring female health workers in rural areas has proven extremely difficult [9]. Areas that have experienced increased insecurity have seen a drop in the number of female health workers who have left the health workforce due to fears of reprisals and recriminations [1]. As community health workers are volunteers, women will remain in the position for as long as they have the time and the desire and most will leave the role after they are married [5].

While access and utilisation of family planning services remains low there have been some positive interventions working within the sociocultural and religious context of communities. Religious leaders, who had been given accurate information about contraceptives and the benefits for women and their families, promoted birth control or birth spacing and played a key role in changing community behaviours [44]. Another study found that medical misconceptions about the safety and efficacy of contraceptives had a bigger impact on uptake then cultural and religious barriers [45]. Once the religious leaders participating in the study had been educated about the benefits they started endorsing birth spacing within the community [45].

The MOPH launched a National Strategy for Improving Quality in Health Care in 2011 to tackle the concerns about care quality in the BPHS which lower utilisation of health care services [46]. These quality improvement interventions were focused on pregnancy and childbirth and have led to improvements in service delivery, engagement with services and knowledge amongst female patients in the study areas [46].

### Affordability

Household out-of-pocket expenditures on health constitute a major barrier to accessing healthcare. High costs and the inability to afford treatment was the reason that 50 % of Afghani survey respondents in 2004–5 gave for not seeking treatment [47]. In 2004 almost 65 % of the total expenditure on health in Afghanistan was incurred as out-of-pocket payments by households [48]. This rose to between 72 % and 79 % of the total expenditure on health in 2006 [7]. Around 40 % of out-of-pocket health expenditures were spent on drugs and supplies, followed by transportation costs (19 %), and consultation fees (14 %) [7]. The World Bank estimated that, in 2014, out-of-pocket expenditure was 63.9 % of the total health budget, falling from 73.3 % in 2011 [49]. We can surmise from this data that out-of-pocket expenditures still finance the majority of health expenditure but the exact figures are unknown as the data used for these studies are incomplete [7]. Out-of-pocket expenditures pose a large financial burden, particularly for the poorest households. Two studies found that the those in the poorest quintile have higher health expenditures then those in the wealthiest quintile [7, 50]. This is perhaps because health needs are greater among the poorest quintile, they postpone seeking care, they live further away and have higher transportation costs.

Transparency International’s corruption index ranks Afghanistan fourth from bottom just ahead of Sudan, North Korea and Somalia [51]. There is a distinct lack of research regarding corruption in the health sector, however the little evidence available suggests it pervades the BPHS, driving up hidden costs for patients and providing a major barrier to accessing healthcare for those who cannot afford the under-the-table payments often required to pay for health services [7, 9].

Another major reason for financial barriers to accessing healthcare is the dominance of an unregulated private health sector. Costs can be punitively high whilst quality is unpredictable, due to insufficient regulation [50]. According to the Afghanistan Health Survey, 57 % of individuals seeking care consult a private provider first, 67 % would visit a private provider for a second visit and 83 % for a third [52]. This finding was supported by a survey conducted in 2008 which found that 75 % of health care visits were to private providers [53]. Provision of medicine is also dominated by the private sector, who sell 70–80 % of all drugs [54]. A study by Trani et al. [50] shows that lack of accessibility to health centres and hospitals had a clear association with seeking private healthcare. Whilst the poorest members of Afghani society have a more positive perception of BPHS facilities than private facilities they consider the former to be less available [54]. Another factor is that the quality of the BPHS services is not consistent and inadequate public funding has driven up user charges [9].

#### Have there been any attempts to overcome this barrier?

The most important step in combatting financial barriers to accessing healthcare in Afghanistan has been the removal of user fees in BPHS facilities, implemented in 2008 [15]. Theoretically, this should go some way to overcoming the difficulties imposed by out-of-pocket expenditure, although limited data currently exists on the full effect of the user fee ban. A study conducted by Steinhardt et al. [15], using data from a 2005 to 2007 health financing study and health facility administrative data from 2008, found that there was no change in observed and perceived quality across fee charging and non-fee charging facilities but that utilisation increased by up to 400 %.

Private sector fees must also be confronted if these barriers are to be overcome. A cap has been introduced on what the private sector can charge, although this has not been enforced systematically [47]. If monitoring and evaluation systems were successfully expanded to include private providers, more data could be gathered and appropriate action could be taken. Until the issue of private sector fees is confronted fully, financial barriers will remain central to access issues.

One lacunae that has not been provided with a convincing solution is the MOPH’s problem of under-spending. Clearly there is no shortage of places where the money could be spent, yet bureaucracy, corruption and understaffing of MOPH offices prevent resources from reaching places where access to healthcare could be improved [9]. The paradox of spiralling budgets and under-spending harms both the credibility and the functioning of the BPHS. Officially the MOPH puts the rate of ‘budget execution’ at around 60 % and states that improving ‘absorptive capacity’ is one of the critical challenges for the health sector, however, they do not set out any specific plans for how this will be achieved [55].

Policies to reduce out-of-pocket expenditure, such as the user fee ban in the BPHS and the regulation of the private sector, are welcomed. Other problems, such as corruption, under-spending and the long-term sustainability of the BPHS have not been meaningfully confronted. If financial barriers to accessing healthcare are to be overcome then fiscal accountability and oversight will have to be tackled effectively through all future aid funding to healthcare, irrespective of the short-term political consequences.

### Availability

The majority of Afghanistan’s population live in rural areas, an estimated 76 % of the total population, and health service availability could be summarised according to a “rural–urban divide” [56]. However, the reality is more complicated and the extent of services available differs by location according to a number of different factors.

Newbrander et al. [1] estimated that there had been a 70 % increase in the number of active BPHS facilities between 2004 and 2011 and that there had also been an increase in utilisation with the number of BPHS facilities seeing more than 750 new patients a month increasing from 22 % in 2004 to 85 % in 2008. Figures for BPHS contracting coverage are also high with the Afghanistan Research and Evaluation Unit estimating in 2006 that 82 % of the country had access to health services [6]. The authors of this report concede that this is probably an overestimation of access as it is a measure of the area that health services cover and the number of people that live in that area [6]. Another indicator of access is to measure the proportion of Afghanis living within an hour’s walking distance of a health facility, a figure that has risen from 9 % in 2002 to 57 % of the population in 2014 [57]. Achieving these high coverage rates has been one of the triumphs of the BPHS, especially given the dire state of the health system before 2003. However, it has been suggested that these figures are misleading as they do not take quality of services into account. Several studies would suggest that the inferior quality of health services is a major issue as some clinics suffered from a lack of staff, drugs, equipment or services and respondents would avoid attending these clinics [48, 58, 59].

Maternal mortality rates (MMR) are estimated by the UN group to have fallen by 64 %, from 1100 per 100,000 live births in 2000 to 396 per 100,000 live births by 2015 [39]. Although this is a modelled mortality estimate and is constrained by data coverage limitations, Akseer et al., [39] suggest that on balance it is likely that the maternal mortality rates have fallen due to the increased availability of maternal interventions. Despite the improvements in coverage of maternal health services there are still large regional disparities. Maternal mortality rates are much higher in the remote, rural areas of the country such as the Ragh district of Badakhshan with an estimated MMR of 650 per 100,000 live births [32]. Carvalho et al. [17] partly ascribes this difference to the much lower coverage of prenatal care (64.1 % in Kabul, 0.6 % in Kandahar and 3.7 % in Badakhshan) and differences in coverage of family planning methods (25.7 % in Kabul, 2.3 % in Badakhshan).

Universality can be considered one of the founding principles of the BPHS. Every citizen of Afghanistan has the right to use its services and government documents outline the extent of services each contracted NGO has to provide. However, in practice there are large disparities in the health workforce between rural and urban areas. The health work force in the rural areas is 16.7 workers per 10000 people compared to urban areas, where the most qualified professional staff are located, with 36 health workers per 10000 people [14].

One of the chief reasons for problems in accessing healthcare in rural areas is the lack of effective infrastructure and transport, compounded by the insecurity of many of the routes. As various communities and clinics have greater distances separating them in rural areas, the need for good transport and infrastructure is greater. Inadequate transportation is a contributing factor to maternal mortality rates as delayed access means patients present with more serious complications resulting in higher fatality rates [32]. A study by Carvalho et al. [17] found major urban–rural disparities; 60 % of people had access to transport from their home to an emergency obstetric clinic in Kabul, whereas in rural Badakhshan this figure was between 5 % and 20 %. Even when the study looked at referral to an emergency obstetric clinic from a health centre rather than from home, 70 % had available transport in Kabul, whereas only 10 % and 30 % had transport in Badakhshan [17]. International donors, such as USAID, have invested heavily into road building projects which should have improved overall transport networks, however, in practice it has been found that there have been a number of drawbacks [60]. The roads have not been maintained, have increased access for International Security Forces (ISAF) and become magnets for insurgent IEDs, making it more rather than less difficult and dangerous to travel [60].

Insecure areas of Afghanistan are far less likely to have accessible BPHS facilities. The number of health facilities in Helmand province, notable for its high intensity of conflict, was reported to have decreased by 38 % between 2004 and 2006 [7]. By 2008 it was found that only 10–15 % of the population of Helmand was covered by the BPHS [61]. Unfortunately, there is lack of available data about the impact of on-going conflict on access to healthcare. It is clear, however, that insecurity impacts access to healthcare and that violence has been increasing countrywide since the withdrawal of foreign troops began in 2014 [18]. The existing studies from Afghanistan have produced skewed results by not collecting data from the most insecure districts [22, 24, 52]. With monitoring and evaluation systems performing in this manner, regional or central branches of the MOPH simply cannot know what level of accessibility is being achieved and therefore cannot respond when underperformance occurs.

The nature of the conflict in Afghanistan has meant that health care workers and health care facilities have become integrated into the conflict. They continue to provide health services to both sides of the conflict but by doing so they expose themselves and become a target [62]. There has been an increase in the number of incidents affecting access to health care, with 125 incidents reported in 2015, compared to 59 in 2014 and 33 in 2013 [19]. Threats and intimidation of health personnel constituted the majority of cases [19]. In the last seven months a clinic in Wardak was attacked by the Afghan special forces and two patients and a carer were executed, and the US bombed the Médecins Sans Frontières (MSF) hospital in Kunduz, causing 85 causalities (42 dead and 43 injured) [62].

Insecurity prevents health workers from being recruited, prevents health facilities from providing services and prevents individuals from getting to those facilities, all of which reduce access to healthcare. The MSF hospital in Kunduz was the only trauma care centre for the whole of the north-eastern region and has remained closed since the attack [19]. The clinic in Wardak has reported a drop in patients attending the facility since the attack as people are too frightened to seek health care [62]. Insecurity was reported by respondents as the main barrier to accessing health care in a 2014 study of patients and caretakers at four MSF-supported clinics in Kabul, Helmand, Kunduz and Khost [59]. A study conducted by MSF in 2013 found that the main obstacle to accessing healthcare for around half of respondents (49 %) was related to conflict and insecurity [63].

One underlying reason that access to healthcare is restricted in conflict-afflicted areas is that many NGOs are often reluctant to run operations in the most insecure areas. This is unsurprising when one considers the amount of security incidents involving NGOs, from January to May 2016 there were 80 NGO incidents including 18 abductions and four fatalities [64].

There is no doubt that NGOs are sometimes seen as being part of the government and Western agenda [65]. Healthcare systems have been politicised and have been used strategically as a war tactic by both sides of the conflict. This has contributed to the shrinking of humanitarian space and compromises an NGO’s ability to remain neutral, impartial and independent [66]. The use of humanitarian assistance as part of the ‘hearts and minds’ campaign by US led Coalition forces led to several incidents of healthcare workers and facilities being targeted by the opposition [67]. The position of these opposition groups toward healthcare has remained in flux. The Taliban have previously released several statements in support of the polio eradication programme, however, in 2015 there were 22 incidents directly affecting vaccination campaigns, mainly attributed to ‘anti-government elements’, including the Taliban [19]. Incidents against health care perpetrated by ‘anti-government elements’ increased by 41 % between 2014 and 2015 [62]. Daesh, which controls areas of Nangarhar, targeted clinics and medical workers reportedly causing 11 clinics to close due to intimidation, looting and extortion [62]. There is no doubt that the BPHS’ ethos of government ownership and priority setting with NGOs involved in service provision has in many instances harmed NGOs by allowing them to be seen as partisan to the government’s and Western policies. This raises questions about NGO independence and ‘rationalises’ attacks on health facilities, further reducing access to healthcare.

#### Have there been any attempts to overcome these barriers?

Mobile health teams have been a recent addition to the BPHS, and are used to reach rural communities [14]. These health teams include one female health provider, one male health provider, a driver and a vaccinator, together ensuring that underserved areas will achieve better access to care [14]. Although they will not be able to fulfil all of the same tasks as a BPHS clinic, they will be able to provide essential drugs and encourage care-seeking behaviour [14]. There is a dearth of literature on the impact of mobile health teams and we could not find any peer reviewed studies that assessed the impact of mobile health teams.

Rural areas in Afghanistan need health services the most and yet have the least access. Poor roads, poverty and insecurity contribute to this problem and remain extraordinarily challenging issues. The introduction of mobile health teams is promising and as yet there is insufficient data to fully evaluate the service. Incentive schemes have shown partial success, Ameli and Newbrander (2008) found that offering incentives for female health workers was successful in rural areas, but not in the most insecure areas [68]. However, they are not adopted by every NGO and should perhaps be expanded to include all underserved rural areas. If geographical barriers to accessing healthcare are to be overcome, there must be more done to end fragmented services provided by various NGOs and bridge the urban–rural divide.

There have been efforts to circumvent the problem of health facilities being targeted. The ‘National Immunization Day’ is an example of a successful delivery method, used for polio vaccines and albendazole (antiparasitic) tablets, with many factions agreeing a ceasefire so that more communities can access these preventive services [69]. Médecins Sans Frontières (MSF) took the decision to opt out of providing services via the BPHS in order to maintain their commitment to neutrality [65]. MSF were then able to convince the Taliban to allow them to provide healthcare in the areas which they controlled [65]. Furthermore, the Taliban realised that allowing MSF to provide services would gain them support from the local population [65]. Other NGOs providing BPHS services have also taken a similar course of action, by distancing themselves from the government and mediating with a community *shura (consultation)* who may be in contact or indeed affiliated with the armed opposition. In this case, the BPHS’ method of contracting is a major strength as NGOs provide an intermediary between communities and the government’s mandate to provide services. If an improvement is to be seen over the next few years, more needs to be done to circumvent or remove the barriers to access imposed by conflict through sustainable negotiated non-aggression and non-interference pacts with the opposition groups that control these areas.

### Limitations

One of the main limitations of analysing the BPHS is insufficient, poor quality and unreliable data. Information presented by key stakeholders must be approached with caution as they have their own interests to protect. NGOs have an interest in proving performance to avoid jeopardising their contracts; structures within the Afghan government have an interest in proving that the money they receive from donors is well spent so that the aid continues; donors have an interest in proving that the Afghan nation-building project was worth the vast amount of resources and people that have already been deployed. Insecure regions are often excluded during the data collection process for safety reasons, which serves to produce a persistent bias that probably contributes to overly positive country averages [63]. Estimates of coverage are also overestimated by the use of crude methods and then quoted widely in government reports. This poor quality data is then recycled by short-term technical advisors who do not have the time to critically evaluate the data provided to them [9].

Many factors which restrict access to healthcare, such as corruption, “ghost workers” and absenteeism are challenging to measure because of their illegality or unacceptability. Perceived wrongdoing could be because of actual wrongdoing, but could also be because of widespread cynicism and distrust in a difficult setting.

Another limitation has been the time lag between recording of data and its publication. For example, a study by Bartlett et al. [33] is based on data collected between 1999 and 2002, up to six years before it was published. This provides a great difficulty in understanding the effect of more recent interventions such as the removal of user fees in 2008 or the introduction of mobile health teams as part of the BPHS in 2010, as not all of the data have become available. For these reasons it is in many ways too early to tell whether the BPHS has done enough to overcome barriers to accessing healthcare. However, too many of the indices and trends are either static or getting worse. There is clearly an urgent need to have a comprehensive and critical review of future policies in delivering health to Afghanistan’s diverse and complex population.

## Conclusions

It is clear that gender inequality, rising insecurity, poor regulation of the costs of accessing healthcare, and the disparity in service provision between rural and urban areas are key barriers to accessing healthcare in Afghanistan.

Policies to address these obstacles have so far been insufficient, some of which have been ameliorative in their effect rather than transformative. Female community health workers are being trained and government commitments have been made towards gender equality, yet these fail to alter the central problem regarding attitudes towards gender. Negotiation with community *shuras* and national days of immunisation may help ease the difficulty of acute delivering healthcare in an insecure setting, but do little to reduce the issue of creating sustainable, secure health delivery. The user fee ban could be a positive step in making health accessible to those who cannot afford it, but does nothing to tackle corruption and the high costs imposed on many by the private sector. Increasing the scope of services offered by the BPHS is a positive step in increasing access to healthcare, but spiralling costs make it less likely that the BPHS will be sustainable without substantial donor funds. Mobile health teams may be a positive step towards bringing healthcare to remote communities, but does little to alleviate poverty and provide general infrastructure which make those communities inaccessible in the first place. An understanding of the barriers to access and the policies that have been implemented to address is critical for Afghanistan to achieve Universal Health Coverage (UHC).

### Future challenges

One of the key factors that may threaten the sustainability of the BPHS in future is spiralling costs. The government has pledged to not only add more services but also expand coverage. Nomads, prisoners and internally displaced persons have been added onto the list of groups entitled to BPHS services [14]. On one hand, vulnerable populations are receiving better *potential* access to healthcare, but on the other the hand the BPHS seems to be growing at an unsustainable rate. The most recent cost estimations of the BPHS are from 2010 and earlier so the current costs are unknown.

When one considers the total absolute costs rather than the per capita cost, the increase is far clearer, starting at 163.6 million USD in 2003, rising to 193.1 million USD in 2005 and 277.7 million USD in 2008 [7]. On a more positive note, when the per capita costs of Afghanistan’s BPHS are compared against previous predictions that have been made, they appear far more balanced. In 1993 the World Bank’s Development Report estimated a similar basic package would cost, in today’s value, roughly 20 USD per capita [70]. More recently, the World Health Organisation (WHO) gave an estimation of 44 USD per capita [71]. Despite costs comparing favourably with estimates, the high level of household out-of-pocket expenditures suggest that per capita costs are an underestimation of real health costs.

The majority of funding for the BPHS comes from three main donors - the World Bank, the European Commission and USAID [11]. Donor funding accounted for 85 % of government expenditure on the BPHS from 2002/03 to 2007/08 [7]. At the beginning of the BPHS support and aid from the donor community into Afghanistan was high and remained so until 2007 [72]. Donor aid then began to plateau between 2008 and 2011 [72]. Contributions from USAID have fallen from US$4.5 to $1.8 billion between 2010 and 2012 and the US Congress announced in 2014 that it intends to halve development aid to Afghanistan in the coming years [63]. Donor funding is not intended to be the main source of income for the health system in the long term [73]. The Ministry of Public Health has set out its’ options for securing funding for BPHS from 2012 to 2020 and concedes that it cannot rely solely on donor funds [55]. Alongside securing sustainable external funding and enhancing aid effectiveness, the Ministry also proposes to harness increased funds from taxation, improve efficiency in public spending and introduce social health insurance [55].

The implementation of the BPHS is achieved through a contracting mechanism between donors, the MOPH and NGOs. It is argued that the ‘least-cost’ approach that is favoured when awarding contracts directly contributes to a loss of quality of care [9]. Some NGOs have been accused of rent-seeking by importing the cheapest drugs and charging for a more expensive version, of retaining salaries to accumulate interest or pocketing funding for health centres that have been closed due to insecurity [9]. A contracting mechanism which is cost-effective is welcome, but not one which compromises quality at the same time. With underperforming and budget ‘top-ups’ taking place, the actual stated budget of the BPHS is an underestimation of the true cost. If, in the distant future, the BPHS is to become sustainably funded by an unaided Afghan treasury, the real cost of the BPHS must be calculated and built into future Ministry planning.

## Abbreviations

BPHS: Basic Package of Health Services
CDC: Centers for Disease Control and Prevention
ISAF: International Security Forces
MMR: Maternal mortality rates
MOPH: Ministry of Public Health
MSF: Médecins Sans Frontières
NGOs: Non-Governmental Organisations
UHC: Universal Health Coverage
UNAMA: United Nations Assistance Mission in Afghanistan
UNDP: United Nations Development Programme
USAID: United States Agency for International Development
WHO: World Health Organisation
